# Evaluation of the Changes in Optical Properties of Peaches with Different Maturity Levels during Bruising

**DOI:** 10.3390/foods10020388

**Published:** 2021-02-10

**Authors:** Ye Sun, Yuping Huang, Leiqing Pan, Xiaochan Wang

**Affiliations:** 1 College of Engineering, Nanjing Agricultural University, Nanjing 210031, China; sunye@njau.edu.cn; 2 College of Mechanical and Electronic Engineering, Nanjing Forestry University, Nanjing 210037, China; huangyuping@njfu.edu.cn; 3 College of Food Science and Technology, Nanjing Agricultural University, Nanjing 210095, China; pan_leiqing@njau.edu.cn

**Keywords:** optical properties, bruise, detection, peach, CLSM spectral absorption (µ_a_) and reduced scattering (µ_s_’)

## Abstract

The main objective was to measure the optical coefficients of peaches after bruising at different maturity levels and detect bruises. A spatially resolved method was used to acquire absorption coefficient (μ_a_) and the reduced scattering coefficient (µ_s_’) spectra from 550 to 1000 nm, and a total of 12 groups (3 maturity levels * 4 detection times) were used to assess changes in µ_a_ and µ_s_’ resulting from bruising. Maturation and bruising both caused a decrease in µ_s_’ and an increase in µ_a_, and the optical properties of immature peaches changed more after bruising than the optical properties of ripe peaches. Four hours after bruising, the optical properties of most samples were significantly different from those of intact peaches (*p* < 0.05), and the optical properties showed damage to tissue earlier than the appearance symptoms observed with the naked eye. The classification results of the Support Vector Machine model for bruised peaches showed that μ_a_ had the best classification accuracy compared to μ_s_′ and their combinations (µ_a_ × µ_s_’, µ_eff_). Overall, based on μ_a_, the average detection accuracies for peaches after bruising of 0 h, 4 h, and 24 h were increased.

## 1. Introduction

Peaches are popular due to their tender texture, appealing flavor, and abundant nutrients [1]. However, due to their thin skin and soft flesh, peaches are easily bruised during postharvest processes, such as harvesting, handling, transporting, and sorting, which have been identified as a major source of reduced fruit quality [2]. The presence of bruising accelerates physiological processes that lead to senescence and spoilage, resulting in a loss of profits for the entire fruit industry [3]. Depending on the extent of damage, it may take up to 12 h for the presence of bruises to become visible, which means that affected produce may not be detected until it reaches the retail store or consumer [4]. In addition, the peel of a peach is composed of multiple colors, part of the skin is dark red, and the pulp that causes internal bruises cannot be detected by the naked eye. Therefore, in the processes of sorting, grading, and sales, the ability to effectively distinguish intact from bruised peaches is of great significance for reducing economic losses.

To date, optical techniques (i.e., multichannel hyperspectral/multispectral imaging, near-infrared spectroscopy) and some other non-destructive technology have been extensively reported and used for the detection of fruit quality [5,6]. Preliminary studies have also demonstrated that these optical technologies can identify underneath bruising in apple [7], blueberry [8,9], avocado [10], and peach [11]. These optical techniques rely on measuring reflected or transmitted light, which is dependent on the system constitute, probe design, lighting setup, and other factors [6]. In optics, fruit is a turbid medium; when the fruit is completely exposed to a light source, the outer surface reflects approximately 4% of the incident light, and the remaining incident energy is transmitted through the surface into the cell structure, and then scattered by small tissue interfaces or absorbed by cellular constituents [2,12,13]. The attenuation of light in turbid tissue is the result of absorption and scattering by the tissue and is represented by the absorption (μ_a_) and reduced scattering (μ_s_′) coefficients [14]. The researcher reported that the absorption and scattering parameters of fruit tissues are related to the material composition and cellular microstructure [15]. However, no studies have been conducted on the effects of peach bruising on optical properties; and it has not been well understood the relationship between the optical properties and the various quality parameters of peaches during bruising.

In the last decade, some studies have been conducted to investigate the effect of bruises on the optical properties of the fruit. Gao et al. [13] investigated the absorption coefficient (μ_a_) and the reduced scattering coefficient (μ_s_′) of kiwifruit flesh by using an integrating sphere system at 950–1650 nm 16 days after bruising. The results showed that μ_s_′ first decreased and then increased, while μ_a_ showed no significant difference resulting from bruising. Lu, Cen, Huang, and Ariana [16] reported that the reduced scattering coefficient of normal apples was much higher than that for bruised apples as shown by spatially resolved hyperspectral scattering images, and it decreased consistently with time after bruising. He et al. [17] developed a spatial frequency domain imaging system (SFDI) to measure the optical parameters of bruised pear, and this method showed perfect results for detecting bruised pears. It can be seen that the changes in optical parameters observed due to bruising in those studies are not the same because the studies use different fruits, detection systems, and sampling methods. However, researchers have not been explored how the optical properties of peaches would change following different periods after bruising. In addition, studies have shown that the maturity of the fruit has a strong influence on the severity of bruise damage [18]. It is important to assess the impact of maturity and the time after bruising on optical properties. Such a report will allow the researcher to understand the complex relationships between quality parameters and optical properties of peach during damage and thus help us improve detection equipment and recognition algorithms.

The aims of this research were therefore to (1) evaluate the effects of maturity stages and detection time points on the optical performance of bruised peach; (2) measure and calculate the reduced scattering and absorption properties of bruised peach; (3) quantify the changes in peach quality parameters (cell structure and color) over time after bruising; and (4) discriminate the bruised peaches with three maturity levels based on the measured μ_a_ and μ_s_′.

## 2. Materials and Methods

### 2.1. Sample Collection

**Figure 1:**
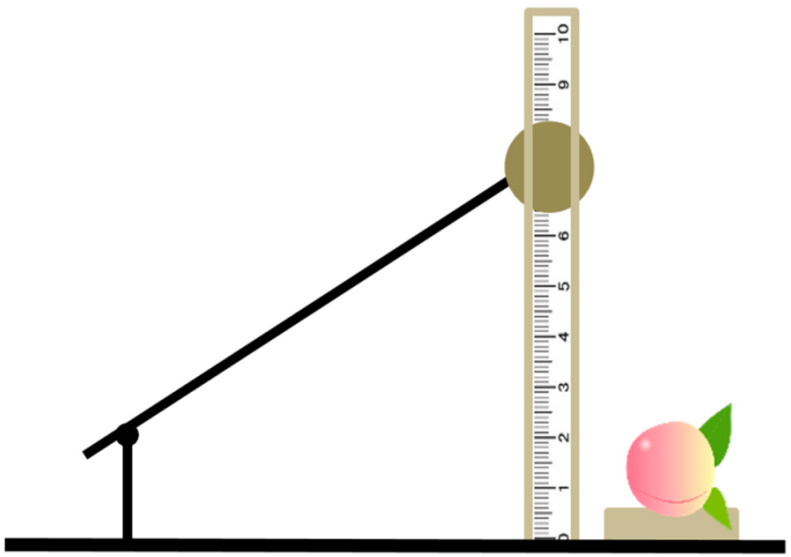
A wooden-ball pendulum impacting device for bruised test on peaches.

The peaches (cultivar of "Redstar") were hand-picked from the orchard of Southwest Research and Extension Center of Michigan State University (Benton Harbor, MI, USA). The pulp of this cultivar was light yellow, while the peel had mixed yellow-red colors unevenly distributed over the surface [19]. After picking, the peaches were transported to the laboratory immediately and placed in the freezer for further research. The sample-set included 330 peaches from three maturity stages assigned based on fruit firmness by an acoustic firmness sensor (AWETA, Nootdorp, The Netherlands), with 110 fruits for each stage (S1: stage 1, acoustic firmness >30, relatively immature; S2: stage 2, acoustic firmness 20–30, commercially ripe; S3: stage 3, acoustic firmness <20, over-ripeness). Among each set, 100 peaches were used for the detection of their optical properties and the remaining 10 peaches were used for the destructive detection of their microstructures. Before the bruising test, peaches from each stage were analyzed as the intact group. After bruising, the samples were analyzed again at three times to monitor the process of bruising: 0 h after bruising, 4 h after bruising, and 24 h after bruising. The bruising tests of peach were manually performed using a wooden ball weighing 161 g with a 74 mm diameter, and the impact energy is changed by adjusting the drop height of the wooden ball. The ball hit the peaches at the equatorial area, as shown in Figure 1. In this research, the impact energy was 0.4 J with a fixed falling height of about 22 mm, which could not clearly observe the damage immediately after bruising. A detailed description of the impact test is given in Zhu et al. [20]. Therefore, there were a total of 12 groups in this research (3 maturity levels × 4 detection times), and the samples were defined as shown in Table 1, each group contained 100 samples.

### 2.2. Image Acquisition and Pre-Processing

**Figure 2:**
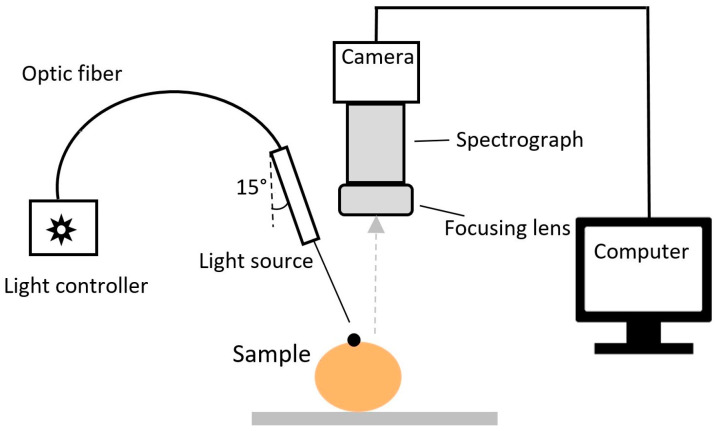
The scheme of a spatially-resolved spectroscopic system for measuring the optical properties of peach samples.

The optical properties of the fruit samples were measured based on the principle of spatially resolved spectroscopy. A detailed description of the principle and parameters of the instrument is given in Cen and Lu [21] and Sun et al. [6]. Figure 2 shows the scheme of a bench-top optical property measurement prototype that was developed in-house based on the spatially resolved spectroscopic principle and coupled with a hyperspectral imaging technique in line-scanning mode to measure the optical properties of the fruit [15,19]. Peaches from each group were measured using this system (Figure 2).

### 2.3. Color Measurement

There is no significant change in the appearance of early bruises, therefore, it is difficult to distinguish the early bruised peach by the naked eye. Hence, a digital colorimeter (CR-400, Konica Minolta Sensing, Inc., Tokyo, Japan) was used for color measurements of pulp and peel of peaches by Lab color model. *L*, *a*, *b* represents Luminosity (value ranges from 0 to 100), the range from magenta to green (value ranges from +127 to −128), the range from yellow to blue (value ranges from +127 to −128), respectively. For the same maturity stage, changes in the peel color reflect the external changes during bruise, and the flesh color reflects the real internal bruise information. After the optical property measurements, the surface color was measured in the bruised area by the digital colorimeter. The diameter of the measurement area and the illumination area of CR-400 were Φ8 mm and Φ11 mm, respectively, and the sample was tested at a standard observation angle of 2°. After calibration, the superficial bruised area of each fruit was measured 3 times, and the averaged values of three measurements were collected for further analysis. Twenty-four hours after bruising, the pulp color was measured after removing a layer of the peel of approximately 5 mm thickness, and the diameter of the bruised area was measured using a Vernier caliper.

### 2.4. Microstructural Analysis

Three fruits from each detection time point of each maturity level were randomly selected for microstructural analysis. Microscopy analysis was conducted on the pulp specimens to observe the microstructural damages of bruises at different maturity levels and detection time stages by Confocal laser scanning microscopy (CLSM, Olympus Fluo View 1000 LSM, Olympus America Inc., Center Valley, PA, USA). CLSM enables us to view a single layer of tissue cells and reduces noise from adjacent layers of cells. Tissue specimens for CLSM were cut from the outer cortex of peaches approximately 10 mm away from the skin.

### 2.5. Data Processing and Analysis

Analysis of variance (ANOVA) and least significant difference (LSD) test was performed on the quality parameters (bruise area, surface color, and pulp color) and optical properties (μ_s_′ & μ_a_) of the peaches at different maturity levels and detection times using SPSS 18.0 Statistics software, which allowed the evaluation of the parameters changes during the bruising. The level of *p* < 0.05 was found significant in all analyses. Bruised fruit were classified by a support vector machine (SVM) and partial least squares discriminant analysis (PLSDA) model using MATLAB 2017a software (The MathWorks, Inc., Natick, MA, USA) based on the optical properties (μ_s_′ and μ_a_) as well as their combinations (i.e., µ_a_, µ_s_’, µ_a_ × µ_s_’, & µ_eff_ = [3µ_a_(µ_a_ + µ_s_’)]^1/2^). The autoscale were applied for data preprocessing of optical properties before modeling. In this research, the type of C-Support Vector Classification (C-SVC) algorithm were adopted, and the radial basis function was selected for kernel type. The calibration was conducted using two-thirds of the samples in a group, and the remaining samples were used for validation.

## 3. Results and Discussion 

### 3.1. Quality Parameter of Bruised Peaches

Changes in the color values of the peel and pulp for different groups of peaches are shown in Table 2. As bruises on peaches are mainly a brown discoloration, the lightness value of L* in the Lab color space is used to represent the color change caused by bruising. Groups 1–3 represent the intact sample at three maturity levels, the peel lightness slightly decreased with the peaches matured, but the only significant difference was found between group 1 and group 3. The peel colors of groups 4–6, 7–9, and 10–12 represent the colors of three maturity stages at each of three time periods (0, 4, and 24 h) after bruising. The lightness decreased over time following bruising as shown in Table 2. But the decrease in lightness not only comes from the browning after bruising but also from the ripening of the fruit. There was no significant difference between immature-S1 and commercially ripe-S2 ones both for intact and early bruised peaches between the following groups: 1 and 2, 4 and 7, 5 and 8, 6 and 9. In other words, for the immature and commercially ripe peaches, it is difficult to distinguish the bruising area from the appearance. For the S3 overripe peaches, the surface skin color was significantly different between the intact group and the corresponding bruised group after 24 h, and the lightness decreased greatly, indicating serious browning. In the early stage after bruising (0, 4 h), most of the skin color was only slightly different from that of intact samples, which means that it is difficult to detect the difference in appearance at the early stage of bruising. Compared with the peel color, pulp had higher L* values for all maturity levels, and the color of the pulp can better express the degree of bruising because the color of pulp is generally milky white and there are fewer interference factors. The color of pulp varied greatly, which means that peaches of different maturity levels suffer different damage when subjected to the same impact energy. The same conclusion can be drawn for the bruised area: the more mature the peach, the larger the bruise diameter (from 10.50 ± 42 mm to 20.37 ± 4.88 mm). The same results were reported in apple, tomato, and loquat; as ripening progresses, tissues lose their cohesion and membrane integrity and, consequently, stress due to impact damage overcomes the cell wall strength, causing bruising [3,22].

**Figure 3:**
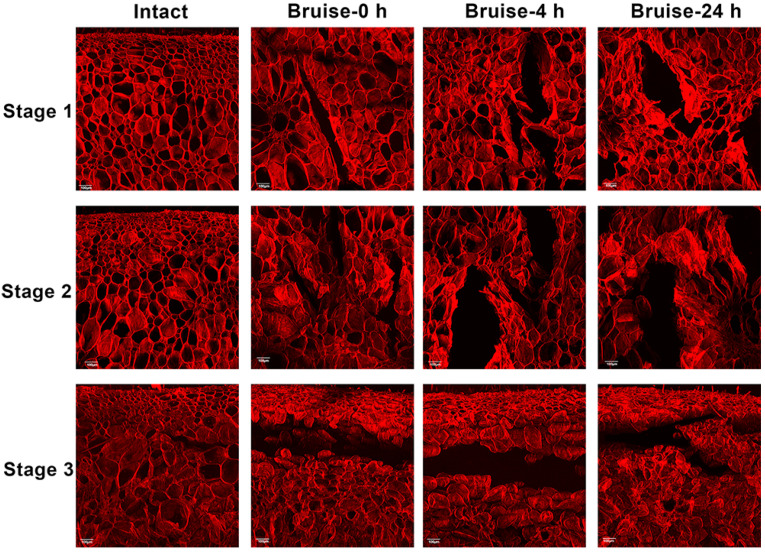
Confocal laser scanning microscopic images of a cross-section of intact and bruised peach flesh tissue for three maturity stages (stage 1–3) and three detection times after bruising (the white ruler shown in the image represents 100 μm).

At an early bruised stage, it is difficult to directly observe bruising by the naked eye, but the damage to the cell microstructure is still significant. Therefore, the effects of maturity and detection time on peach bruising were observed by CLSM, and the images of peach pulp tissues from the twelve groups are shown in Figure 3. In intact tissue of peaches at maturity stages 1 and 2, the cells were arranged neatly and the cell membrane was smooth and intact. For intact tissue of overripe peaches at stage 3, the cell membrane was slightly shrunken, and loose connections were observed between cells. For the S1 samples, the cracks were neat, and the cells around the cracks were evenly distributed; as the time after bruising increased, the size of the cracks increased and the cell membrane shrank. In comparison, the S2 samples had larger cracks and more obvious shrinkage of the cell membrane. S3 samples were significantly different from the S1 and S2 samples, as shown in Figure 3. The cracks ran through the entire image, and the cells were severely shrunken. Moreover, due to the pectin degradation and water redistribution, the cell structure changed, indicating that the cells had aged. These results confirmed that bruising is a result of cell breakage, which is caused by stress and distortion of individual cells [3,23].

### 3.2. Optical Properties of Bruised Peaches

**Figure 4:**
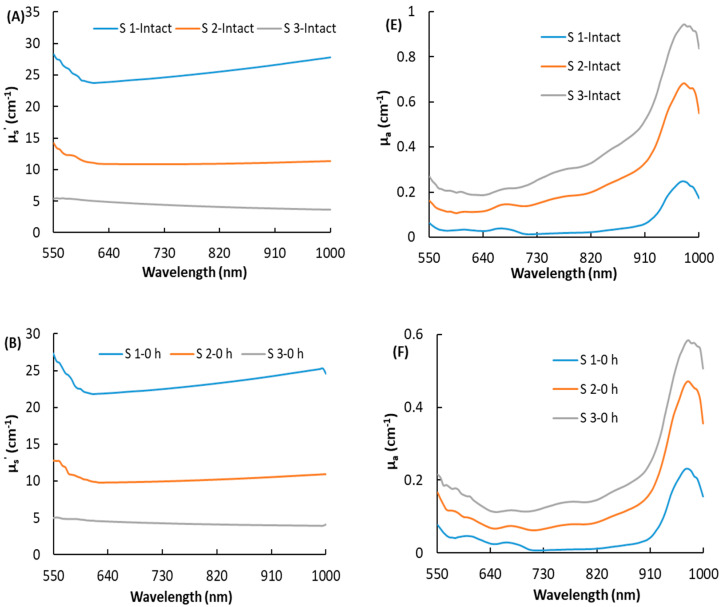

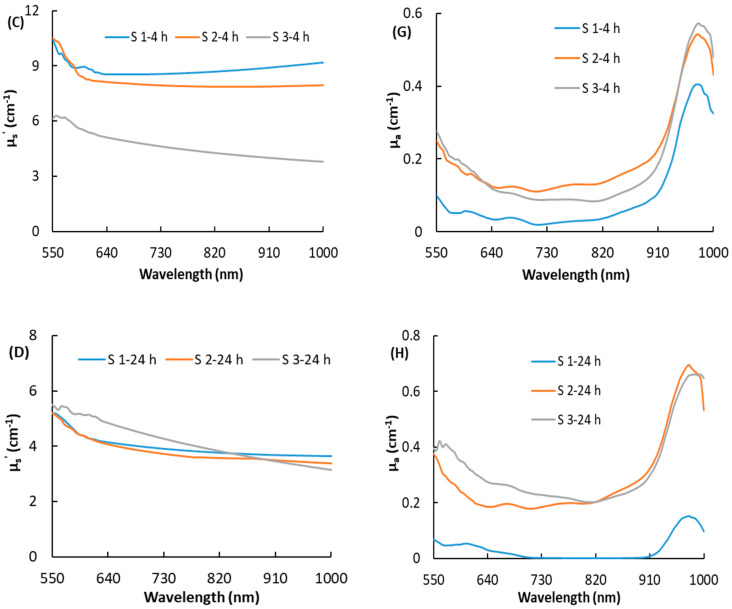
The reduced scattering coefficient (µ_s_’) and the absorption coefficient (µ_a_) of peaches at different maturity stages for health (**A**,**E**), bruise after 0 h (**B**,**F**), bruise after 4 h (**C**,**G**), and bruise after 24 h (**D**,**H**).

Figure 4 shows the effect of maturity on optical properties following different times after bruising. The μ_s_′ curve decreased first and then flattened over 550–1000 nm with no characteristic peaks. For the immature peaches (S1), the μ_s_′ value increased over 550–1000 nm, while for the overripe peaches (S3), the μ_s_′ value decreased over 550–1000 nm. However, the changes in μ_s_′ among the different maturity stages were still quite large; μ_s_′ decreased as the peach matured. Different from μ_s_′, the μ_a_ varies greatly with wavelength, and two absorption peaks were observed around 675 and 970 nm. The prominent peak at approximately 970 nm was ascribed to the second overtone O-H stretching from water [24]. However, the absorption peak at 675 nm, which was reported as the absorption of chlorophyll-a [19], was not obvious in this research. A possible explanation is that this ‘Redstar’ peach has a low chlorophyll-a content in the red skin. The changes in μ_a_ among the different maturity stages were opposite to the changes in μ_s_′, in that μ_a_ gradually increased as the peach matured. It can be seen from Figure 4A, E that for the S1 samples, the values of μ_s_′ were much larger than the values of μ_a_, which means that the diffusion approximation equation is satisfied (μ_s_′ >> μ_a_). As peach overripeness or bruising occurs, they both cause tissue softening, leading to cell wall depolymerization, increased solubility of the gelatin layer, and separation of the cytoplasmic wall. At this time, μ_s_′ rapidly decreased, and the assumption of scattering domination for the diffusion approximation equation may no longer be valid, which means that the calculated values of μ_a_ and μ_s_′ may not accurately reflect the true values of the sample, the same results also reported in Cen et al. [14] and Sun et al. [6]. This is also a possible reason why the spectral curves of μ_a_ in Figure 4G,H were abnormal.

The means and standard errors for the optical properties of two characteristic wavelengths of 675 and 970 nm for the twelve groups of peaches as shown in Table 3. The chlorophyll is reported as an important index to evaluate fruit quality, when peach suffered from environmental adversity such as senescence, impact, or pathogen infection, the chlorophyll content declined [12,25]. In addition, with maturity, there is usually a gradual decrease in the strong absorption peak at 970 nm which is mainly due to water. Table 3 shows the changes in the optical characteristics of peaches during the bruising process, with S1 represented by groups 1, 4, 5, and 6; S2 by groups 2, 7, 8, and 9; and S3 by groups 3, 10, 11, and 12. For the S1 samples, in the first 0 h after bruising, there was a slight change in optical properties, and there was no significant difference between groups 1 and 4, except for μ_s_′ at 970 nm. Four hours after bruising, there were significant differences in the optical parameters between bruised samples (group 5) and intact samples (group 1), but there were no significant differences in the peel color at this time, which show that optical properties can reflect tissue changes from bruising earlier than appearance symptoms can. Therefore, the optical properties have the potential to distinguish bruised peaches at the early stage. A similar conclusion was found for the S2 samples, in that the optical properties of intact samples (group 2) were significantly different from the optical properties of bruised samples 4 h and 24 h after bruising (groups 8 and 9). For the S3 samples, the μ_s_′ of the intact samples (group 4) was reduced to a small value, and the changes in optical parameters were relatively small during the process of bruising, resulting in an insignificant difference between the groups. This may be due to the decreasing density of the scattering particles for an overripe peach, and the particle size may change due to the dissolution of pectin. According to the empirical formula (μ_s_′ = aλ − b), the physical properties of these scattering particles are related to the scattering ability of fruit tissue, where the parameter "a" is proportional to the density of the scattering particles and "b" depends on the particle size [6]. Therefore, due to the decreasing of the density of the scattering particles and the increase of the size of scattering particles in the postharvest stage of ripening, softening, and bruising, μ_s_′ would be expected to decrease.

### 3.3. Classification of Bruised Peaches

**Figure 5:**
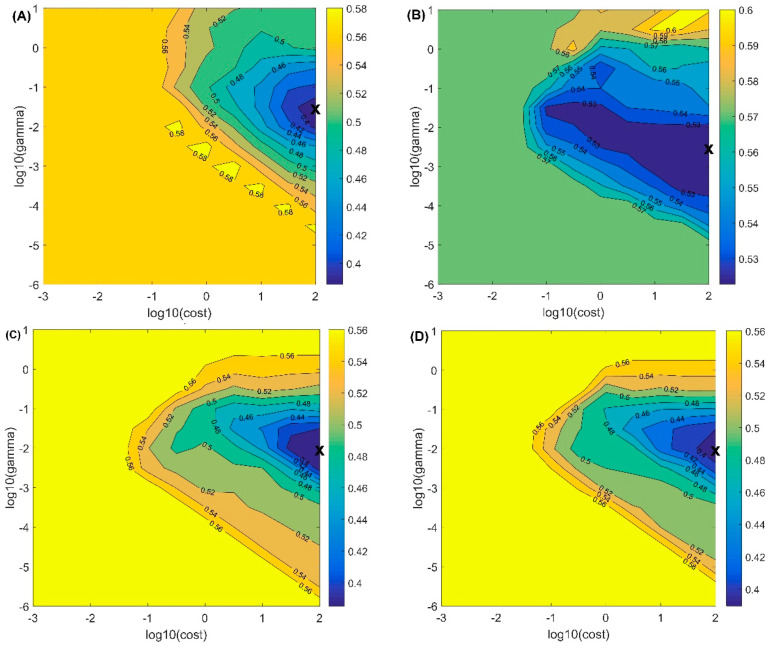
The misclassification fraction of cross-validation for the SVM model based on µ_a_ (**A**), µ_s_’ (**B**), µ_a_ × µ_s_’ (**C**), and µ_eff_ (**D**).

Table 4 shows the SVM results for the bruised peaches using the optical properties as well as their combinations (μ_s_′, μ_a_, µ_a_ × µ_s_’, & µ_eff_ = [3µ_a_ (µ_a_ + µ_s_’)]^1/2^) over 550−1000 nm. The bruise class included samples from groups 4–12 of all maturity stages (900 samples), while the intact class consisted of groups 1–3 (300 samples). Two-thirds of the samples from each class were randomly selected for the training set, and the remaining third was used as the testing set. As shown in Table 4, the classification accuracies ranged between 24% and 93.67% for the tested samples. Comparing the properties, μ_s_′ had a poor overall classification accuracy of 76.25%, while μ_a_ had the highest overall classification accuracy of 85%. These results were quite different from those of some other studies. Researchers have reported that combined data (µ_a_ × µ_s_’, & µ_eff_) usually show better results than single parameters, but the classification accuracies of the combined data in this study were slightly lower than the results of µ_a_. The possible reason for this result is that the maturity levels have a strong influence on µ_s_’. Overripeness and bruising both leads to a decrease in µ_s_’, so the µ_s_’ values of overripe intact peaches partially overlap with the µ_s_’ values of bruised samples, which makes it easy to misclassify an intact overripe peach as a bruised peach. Figure 5 shows the selection method for the two parameters (gamma and cost) in the SVM model based on the misclassification fraction of cross-validation. The conclusions are consistent with the results presented in Table 4. The optimal misclassification fraction of the cross-validation model shown in Figure 5B is the largest among all four models, while the optimal misclassification fractions of the other three models are below 0.4. A simpler algorithm of PLS-DA were used to validate the classification performance of data, as shown in the supplemental material (Table S1), the overall classification accuracy is lower than the results of SVM, and similar results were obtained that μ_a_ had the highest classification accuracies. Overall, the optical parameters showed the ability to identify bruised peach samples for the multiple maturity stages and post-bruising times. However, the classification results of different groups by the optical properties are still unclear. In addition, further research is needed on the effect of maturity stages and times on bruise detection.

Based on the above research, it was found that µ_a_ had the best classification effects for bruise detection. Therefore, μ_a_ was used for further research to classify bruised peaches in different groups via the SVM model based on wavelengths from 550–1000 nm; the classification results are shown in Table 5. Each group contained 100 samples, 67 samples of each group were randomly selected for the training set, and the other 33 samples were used as the testing set. The classification accuracies ranged between 57.35% and 100% for the tested samples. The lowest classification results were observed between group 1 (S1-intact) and group 4 (S1—0 h after bruising), which means that the occurrence of bruising will not immediately cause a very large change in the optical parameters of the tissue when the peach is immature with a hard texture. The highest classification accuracy of 100% was observed between group 1 and group 9 (S2—24 h after bruising), group 1 and group 11 (S3—4 h after bruising), and group 1 and group 12 (S3—24 h after bruising), which means that with the dual effects of peach ripening and bruising, the optical absorption coefficient changes significantly. With the extension of the bruising time, the classification accuracy between the intact group and the corresponding bruised group gradually increased. For S1 peaches, the detection accuracy increased from 57.35% at 0 h after bruising to 94.12% at 24 h after bruising; for S2 peaches, from 91.18% to 95.59%, and for S3 peaches, from 83.82% to 94.12%. Researchers have reported that as time passes after bruising, damage to cell tissue initiates contact between the polyphenol oxidase (PPO) and peroxidase (POD) cytoplasmic oxidizing enzymes and phenolic contents originally stored in the vacuole [26]. Enzymatic oxidation in damaged cells converts phenolics into quinones, which polymerize to form dark/brown pigments on the damaged part of the fruit [26,27]. Therefore, the more time that passes since damage occurs, the higher the detection accuracy, and therefore, it is difficult to detect bruise damage at an early stage. In addition, for the peaches from different maturity stages (for example bruised S3 and intact S2, bruised S1, and intact S3), the classification results were irregular during the extend of the bruising time. The possible reason is that maturity also leads to changes in optical characteristics when the difference in maturity was higher than the difference caused by bruising, the detection results were high even in the early stages of bruises.

## 4. Conclusions

In this research, the spectral absorption (µ_a_) and reduced scattering (µ_s_’) coefficients spectra were obtained from 550 to 1000 nm for bruised peaches. The absorption spectra gradually decreased during maturation and bruising, while the reduced scattering spectra showed an increasing trend. The two absorption peaks of µ_a_ spectra were observed at approximately 675 nm for chlorophyll-a and 970 nm for water, while the μ_s_′ curve first decreased and then flattened over 550–1000 nm with no characteristic peaks. Mature peaches are more susceptible to outside pressure of bruise damage than immature peaches, while the optical properties of immature peaches changed more after bruising than the optical properties of ripe peaches. The Support Vector Machine (SVM) model were adopted for classification, the classification accuracy for intact and bruised peaches based on μ_a_, μ_s_′, µ_a_ × µ_s_’, and µ_eff_ was 85%, 76.25%, 84.75%, and 84.5%, respectively. The detection effects were further analyzed based on μ_a_ for 12 subgroups (3 maturity levels * 4 detection times), with classification accuracies from 57.35% to 100%. The highest classification accuracy of 100% was observed between intact immature peaches and 24 h bruised peaches, which means that with the dual effects of peach ripening and bruising, the optical absorption coefficient changes significantly. These results provide a basic understanding of the optical characterization of peaches under bruising.

## Figures and Tables

**Table 1:** Grouping introduction of the total twelve groups *.

Maturity	Intact	Detection Time		
		0 h	4 h	24 h
S1	Group 1	Group 4	Group 5	Group 6
S2	Group 2	Group 7	Group 8	Group 9
S3	Group 3	Group 10	Group 11	Group 12

* S1: stage 1, relatively immature; S2: stage 2, commercially ripe; S3: stage 3, over-ripe.

**Table 2:** Means and standard deviations of the quality parameters for different groups of peaches *.

Groups		Peel Color (L* Value)	Pulp Color (L* Value)	Bruises Diameter (mm)
Intact	1	49.38 ± 7.10 ^f^	/	/
	2	46.75 ± 9.28 ^e,f^	/	/
	3	45.53 ± 10.02 ^c,d,e^	/	/
S1-bruise	4	47.63 ± 5.05 ^e,f^	/	/
	5	47.10 ± 7.17 ^e,f^	/	/
	6	45.95 ± 4.71 ^d,e^	67.26 ± 5.50 ^a^	10.5 ± 5.42 ^a^
S2-bruise	7	46.51 ± 6.17 ^e,f^	/	/
	8	44.61 ± 6.90 ^c,d,e^	/	/
	9	43.04 ± 7.96 ^c,d^	56.14 ± 11.62 ^b^	13.96 ± 4.94 ^b^
S3-bruise	10	42.88 ± 5.29 ^c^	/	/
	11	37.89 ± 7.67 ^b^	/	/
	12	35.13 ± 8.59 ^a^	47.11 ± 6.98 ^c^	20.37 ± 4.88 ^c^

* Data were expressed as the mean of 100 samples ± standard deviation. Numbers on the same column with different letters are different (*p* < 0.05). ‘/’ represents uncollected data.

**Table 3:** Means and standard deviations of the optical parameters for different groups of peaches *.

Groups		µ_a_ 675 nm	µ_a_ 970 nm	µ_s_’ 675 nm	µ_s_’ 970 nm
Intact	1	0.04 ± 0.04 ^a^	0.24 ± 0.26 ^a^	24.64 ± 8.80 ^e^	27.86 ± 10.14 ^g^
	2	0.09 ± 0.10 ^b,c^	0.34 ± 0.36 ^b^	15.79 ± 7.61 ^d^	14.74 ± 10.15 ^d^
	3	0.20 ± 0.07 ^e^	0.73 ± 0.20 ^e^	4.96 ± 0.72 ^a^	3.85 ± 0.67 ^b^
S1-bruise	4	0.03 ± 0.03 ^a^	0.23 ± 0.21 ^a^	22.85 ± 9.52 ^e^	25.23 ± 9.83 ^f^
	5	0.09 ± 0.10 ^b^	0.54 ± 0.27 ^c^	8.65 ± 6.42 ^b^	8.73 ± 6.65 ^e^
	6	0.11 ± 0.11 ^c,d^	0.58 ± 0.31 ^c^	4.01 ± 0.98 ^a^	3.88 ± 0.99 ^b^
S2-bruise	7	0.07 ± 0.04 ^b^	0.34 ± 0.25 ^b^	13.90 ± 7.83 ^c^	16.90 ± 7.83 ^e^
	8	0.11 ± 0.11 ^b,c,d^	0.53 ± 0.25 ^c^	7.05 ± 6.21 ^b^	7.05 ± 6.21 ^c^
	9	0.14 ± 0.08 ^d^	0.59 ± 0.20 ^c^	3.84 ± 0.87 ^a^	3.74 ± 0.87 ^b^
S3-bruise	10	0.21 ± 0.12 ^e^	0.75 ± 0.07 ^e^	4.88 ± 2.65 ^a^	4.41 ± 0.65 ^b^
	11	0.22 ± 0.12 ^e^	0.74 ± 0.27 ^e^	4.60 ± 2.13 ^a^	3.97 ± 1.94 ^b^
	12	0.27 ± 0.17 ^f^	0.68 ± 0.27 ^e^	4.33 ± 1.00 ^a^	3.22 ± 0.91 ^a^

* Data were expressed as the mean of 100 samples ± standard deviation. Numbers on the same column with different letters are different (*p* < 0.05).

**Table 4:** Classification results for bruised peach samples via Support Vector Machine (SVM) model using optical parameters (μ_a_, μ_s_′, μ_a_ × μ_s_′, and μ_eff_) over 550–1000 nm *.

Optical Parameter	Actual Class	Model Parameters	Training Set (%)	Testing Set		
				Predicted Intact Class	Predicted Bruise Class	Accuracy (%)
(A) μ_a_	Intact	Cost: 100 Gamma: 0.0316 Number of SVs: 245	90.00	76	24	76.00
	Bruise		93.50	36	264	88.00
	Overall		92.63	112	288	85.00
(B) μ_s_′	Intact	Cost: 100 Gamma: 0.0316 Number of SVs: 353	22.50	24	76	24.00
	Bruise		96.17	19	281	93.67
	Overall		77.75	43	357	76.25
(C) μ_a_ × μ_s_′	Intact	Cost: 100 Gamma: 0.01 Number of SVs: 251	83.50	62	38	62.00
	Bruise		95.00	23	277	92.33
	Overall		92.13	85	315	84.75
(D) μ_eff_	Intact	Cost: 100 Gamma: 0.01 Number of SVs: 243	85.00	63	37	63.00
	Bruise		94.00	25	275	91.67
	Overall		91.75	88	312	84.50

* The bruise class included samples from groups 4–12 of all maturity stages, while the intact class consisted of groups 1–3.

**Table 5:** Discrimination result between bruised samples and intact samples via Support Vector Machine (SVM) model using μ_a_ over 550–1000 nm.

Discrimination Result (%)					
Bruised Sample		Intact Sample			
Maturity	Detection Time	S1-Intact	S2-Intact	S3-Intact	Overall
S1	0 h-Bruise	57.35	82.35	95.58	78.43
	4 h-Bruise	86.71	79.41	94.12	86.75
	24 h-Bruise	94.12	95.59	94.12	94.61
	Overall	79.39	85.78	94.61	/
S2	0 h-Bruise	79.41	91.18	95.58	88.72
	4 h-Bruise	98.53	94.12	94.12	95.59
	24 h-Bruise	100	95.59	94.12	96.57
	Overall	92.65	93.63	94.61	/
S3	0 h-Bruise	98.53	82.35	83.82	88.23
	4 h-Bruise	100	79.41	86.77	88.72
	24 h-Bruise	100	95.59	94.12	96.57
	Overall	99.51	85.78	88.24	/

## Supplementary Materials

The following are available online at https://www.mdpi.com/2304-8158/10/2/388/s1, Table S1: Two-class classification results for the training and test sets of peach samples via partial least squares discrimination analysis (PLS-DA) model using optical parameters (μa, μs′, μa × μs′ and μeff) over 600–1000 nm.
