# Supplementary material for: Evaluation of the Changes in Optical Properties of Peaches with Different Maturity Levels during Bruising
Source: Foods. 2021 Feb 10;10(2):388. doi: 10.3390/foods10020388 (PMC7916705; doi:10.3390/foods10020388)
Supplement: Supplementary file 1 [file foods-10-00388-s001.pdf]

Supplementary Table S1. Two-class classification results for the training and test sets of peach samples via partial least squares discrimination analysis (PLS-DA) model using optical parameters (μ_a, μ_s', $\mu_a \times \mu_s'$ and μ_{eff}) over 600–1000 nm.

**Optical parameter**	**Actual class**	**Training set (%)**	**Testing set**		
			Predicted intact class	Predicted bruise class	Accuracy (%)
**(A)** μ_a	Intact	90.50	94	6	94.00
	Bruise	75.75	44	156	78.00
	Overall	79.44	98	202	82.00
**(B)** μ_s'	Intact	39.50	42	58	42.00
	Bruise	76.75	49	151	75.50
	Overall	67.44	34	266	67.13
**(C)** $\mu_a \times \mu_s'$	Intact	87.00	79	21	79.00
	Bruise	83.75	32	168	84.00
	Overall	84.56	81	219	82.75
**(D)** μ_{eff}	Intact	84.50	73	27	73.00
	Bruise	81.50	24	176	88.00
	Overall	82.25	79	221	84.25
